# Extracellular RNAs as Messengers and Early Biomarkers in Neurodegeneration

**DOI:** 10.3390/ijms27010320

**Published:** 2025-12-27

**Authors:** Kaidong Lu, Magdalena J. Koziol

**Affiliations:** 1 College of Biological Sciences, China Agricultural University, Beijing 100193, China; 2 Beijing Institute for Brain Research, Chinese Academy of Medical Sciences & Peking Union Medical College, Beijing 102206, China; 3 Chinese Institute for Brain Research, Beijing, Beijing 102206, China

**Keywords:** extracellular RNA, extracellular vesicles, blood–brain barrier, neurodegeneration, biomarkers, neuroinflammation, mitochondria

## Abstract

Extracellular RNAs are released from cells and circulate stably in biofluids such as blood, cerebrospinal fluid, saliva, and urine via carriers including extracellular vesicles, RNA-binding proteins and lipoproteins. Because transcriptional and metabolic disturbances—notably mitochondrial dysfunction and oxidative stress—often precede protein aggregation, synaptic loss, and structural change in many brain diseases, exRNAs offer minimally invasive access to early disease biology. Mechanistic studies demonstrate selective RNA packaging and delivery: transferred mRNAs can be translated and miRNAs can modulate targets, indicating exRNAs both report intracellular programs and actively influence recipient cells. Clinical and preclinical data support a dual role for exRNAs as biomarkers and as mediators of pathology. Key technical hurdles—pre-analytical variability, isolation heterogeneity, and uncertain cellular origin—limit reproducibility; recommended solutions include standardized workflows, carrier- and cell type-specific enrichment, multimodal integration with proteomics/metabolomics and neuroimaging, and large, longitudinal validation studies. We synthesize mechanistic and clinical evidence for exRNA utility in early detection, prognosis, and therapeutic targeting and outline a roadmap to translate exRNA findings into robust clinical assays and interventions for neurodegenerative and brain disorders.

## 1. Introduction

Extracellular RNAs (exRNAs) encompass a diverse set of RNA species released by cells into the extracellular milieu and detectable in biofluids such as blood, cerebrospinal fluid (CSF), saliva, and urine [1,2,3]. Major classes include microRNAs (miRNAs, ~22 nucleotides), long noncoding RNAs (lncRNAs), circular RNAs (circRNAs), mitochondrial RNAs (mt-RNAs), and messenger RNAs (mRNAs). In bodily fluids, these molecules are often protected. For example, quantitative profiling indicates that more than 90% of circulating miRNAs are associated with Argonaute-2 (Ago2) or other RNA-binding proteins, whereas less than 10% are vesicle-associated [4]. This selective protection not only prolongs RNA half-life but also suggests that exRNAs are biologically meaningful rather than random degradation products [4,5,6]. Stabilized carriers likely protect them from abundant nucleases and enable systemic transport—principally membrane-bound extracellular vesicles (EVs), RNA-binding protein complexes, and lipoprotein particles [3,4,5,6]. There are two types of EVs: exosomes are generated from the endosomal system and released upon multivesicular body fusion with the plasma membrane [7,8,9,10], whereas microvesicles are formed by the direct outward budding and fission of the plasma membrane [8,9,11]. Here, we provide only the general biogenetic context of these vesicles, as detailed subclass distinctions and atypical biogenesis routes are beyond the scope of this review.

Experimental work has established two complementary roles for exRNAs. First, exRNA repertoires mirror regulated intracellular transcriptional and stress responses: selective sorting mechanisms direct specific RNAs into carriers in a manner responsive to neuronal activity, metabolic state, and cellular stress [12,13,14,15,16]. Second, exRNAs can be functional effectors: EV-delivered mRNAs may be translated in recipient cells and EV-shuttled miRNAs can repress target mRNAs, demonstrating that exRNA transfer can modulate gene expression networks in distant cells [12,17]. This duality—that exRNAs both report cellular programs and can alter recipient cell biology—motivates their study as biomarkers and as potential mediators or therapeutic vectors in central nervous system (CNS) disorders.

Given that neurodegeneration is generally chronic and slowly progressive [18,19], early detection and timely intervention during the initial phases of disease development would be of great significance. Neurodegenerative and related brain disorders share early convergent molecular features that are accessible to exRNA-based interrogation. Conditions including Alzheimer’s disease (AD) and its prodrome mild cognitive impairment (MCI), Parkinson’s disease (PD), amyotrophic lateral sclerosis (ALS), frontotemporal dementia (FTD), Huntington’s disease (HD), multiple sclerosis (MS), traumatic brain injury (TBI), and glioblastoma exhibit early transcriptional dysregulation, mitochondrial dysfunction, metabolic changes and oxidative stress that often precede overt protein aggregation, synaptic loss, and macroscopic structural changes [20,21,22,23,24,25,26,27,28,29,30,31,32,33,34,35,36,37,38,39,40,41,42,43,44,45,46,47]. For example, in AD, progressive neuronal loss concentrates in the hippocampus and cerebral cortex with marked synaptic loss [20,21]; brain glucose hypometabolism, mitochondrial dysfunction, and transcriptomic abnormalities including altered transcription and splicing are evident, and aberrant expression of noncoding RNAs (miRNAs, circRNAs, lncRNAs) has been detected in affected regions [20,21,22,23,24,25,26,27,28,29,44]. A similar pattern occurs in PD, where dopaminergic neurons in the substantia nigra pars compacta are lost and neuronal energy and lipid metabolism are altered [30,31], with abnormal metabolites such as lactate [30,31]; dysregulation of miRNAs including miR-7 and miR-153 has been linked to dopaminergic vulnerability [30,31,32,33,45]. Likewise, ALS features progressive upper and lower motor neuron loss, mitochondrial dysfunction and energy deficits, impaired nucleocytoplasmic RNA transport, mislocalization of RNA-binding proteins such as TDP-43, aberrant splicing, and noncoding RNA imbalance [34,35,36,37,38,46,47]. Current clinical diagnostics—for example CSF amyloid-β and tau measures, α-synuclein assays, neurofilament light (NfL), and neuroimaging (magnetic resonance imaging, MRI)—predominantly detect downstream proteinopathy or structural injury and commonly become abnormal only after substantial neural damage [43,48,49,50,51,52]. Detecting earlier molecular disturbances therefore offers the opportunity to identify at-risk individuals, to monitor disease trajectories before irreversible loss, and to enrich clinical trials with patients in stages most likely to benefit from disease-modifying interventions.

exRNAs provide a minimally invasive window into these early transcriptional and metabolic changes because they (i) arise from regulated intracellular programs and stress responses, (ii) can traverse or signal across the blood–brain barrier (BBB) via CSF drainage, transcytosis, and glymphatic/meningeal lymphatic routes [53,54,55,56,57], and (iii) are amenable to sensitive assay in peripheral fluids for screening and longitudinal monitoring [16,58,59,60]. Examples from clinical and preclinical studies illustrate this potential: circulating miRNA panels and EV miRNA signatures discriminate MCI/AD from controls and relate to amyloid/tau pathology [61,62]; exosomal lncRNA BACE1-AS, known to stabilize BACE1 mRNA (encoding the β-secretase essential for pathogenic amyloid-β generation in AD), correlates with amyloid processing and enhances diagnostic performance when combined with imaging [63]; and plasma EV profiling has identified dysregulated transcripts such as PHGDH and mitochondrial genes that appear prior to symptom onset in some cohorts [64,65]. Mechanistic studies further implicate exRNAs as active mediators that propagate neuroinflammation and synaptic dysfunction: for instance, miR-137, miR-21 and miR-146a-5p have been linked to oxidative stress, neuronal death, and synaptic protein downregulation [17,66,67,68].

Translating exRNA discoveries into clinically useful assays faces important technical and conceptual challenges. Pre-analytical variables (biofluid choice, anticoagulant, processing delays, centrifugation steps, freeze–thaw cycles) and heterogeneous isolation methods (total plasma RNA, ultracentrifugation, size-exclusion chromatography, precipitation kits, immunocapture) substantially influence the distribution of vesicular versus non-vesicular pools and thereby affect measured exRNA profiles [13,14,15]. Assigning cellular origin is nontrivial because circulating signals may reflect a composite of CNS release, peripheral responses to CNS pathology, and unrelated systemic processes; immunocapture of neuron-derived EVs (NDEs) using surface markers such as L1CAM, SNAP25 or MAP2 improves CNS specificity but remains limited by antibody specificity and marker shedding [69,70,71]. Finally, small, cross-sectional cohorts and variable normalization strategies have constrained replication and generalizability.

In this review, we synthesize the mechanistic and clinical evidence positioning exRNAs as both early biomarkers and active mediators in neurodegenerative and brain disorders. We describe exRNA biology and trafficking across the BBB, summarize disease-specific findings while preserving reported gene and transcript details, and emphasize mitochondrial and oxidative stress-linked exRNA signatures as representative early molecular changes. We then discuss technical considerations for rigorous biomarker development, outline translational and therapeutic opportunities, and recommend a roadmap—standardized pre-analytical standard operating procedures (SOPs), carrier and cell type enrichment, multi-omics integration with proteomics/metabolomics and neuroimaging (including MRI), and large, longitudinal, diverse cohorts—to enable validation and clinical deployment of exRNA-based diagnostics and interventions.

## 2. Biology of Extracellular RNAs

**Figure 1:**
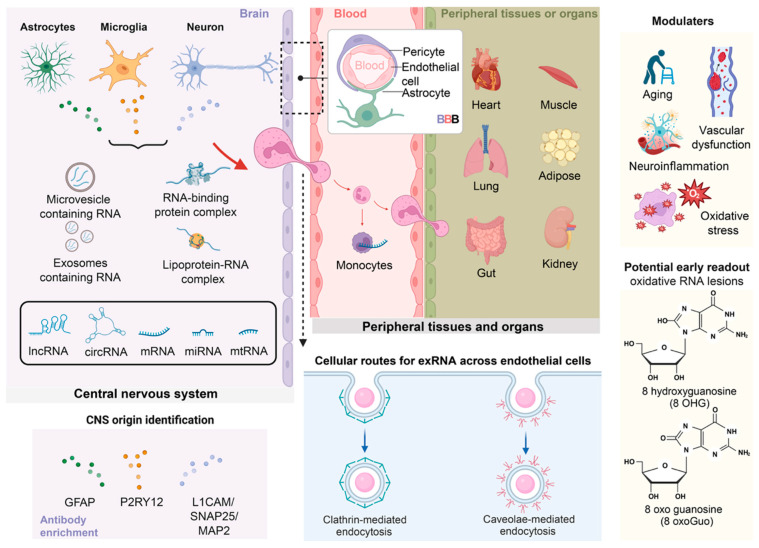
Overview of extracellular RNA (exRNA) species, carriers, and routes linking the central nervous system (CNS) to peripheral organs. Cells in the CNS, including astrocytes, microglia, and neurons, release diverse exRNA species such as microRNAs (miRNAs), long noncoding RNAs (lncRNAs), circular RNAs (circRNAs), messenger RNAs (mRNAs), and mitochondrial RNAs (mtRNAs) [2,72]. These RNAs are transported via distinct extracellular carriers, including membrane-bound extracellular vesicles (EVs; small extracellular vesicles (sEVs)/exosomes and larger microvesicles), RNA-binding protein complexes (e.g., Argonaute-2), and lipoprotein-associated RNA particles (e.g., high-density lipoprotein, HDL) [1,2,3,4,5,6,12,17,64,72]. Within the neurovascular unit, endothelial cells, pericytes, and astrocytic endfeet form the blood–brain barrier (BBB), through which exRNAs may traverse via clathrin-mediated or caveolae-mediated endocytosis [73]. Cell type-enriched markers (e.g., GFAP for astrocytes, P2RY12 for microglia, L1CAM/SNAP25/MAP2 for neurons) enable identification of CNS-derived exRNAs in peripheral fluids [69,70,71,74,75,76]. Once in circulation, CNS-origin exRNAs can reach peripheral tissues and organs such as the heart, lung, muscle, adipose tissue, gut, and kidney [77,78,79]. Systemic modulators, including aging, vascular dysfunction, neuroinflammation, and oxidative stress, shape both the release and peripheral detectability of exRNAs [53,54,55,56,57,80,81,82]. Oxidative RNA lesions such as 8-hydroxyguanosine (8-OHG) and 8-oxo-guanosine (8-oxoGuo) represent potential early readouts associated with CNS dysfunction [83]. Solid arrows indicate established biological processes involved in extracellular RNA (exRNA) release, transport, and intercellular communication. Dashed arrows denote known cellular routes by which exRNAs traverse endothelial cells, illustrating established transcellular pathways rather than hypothetical mechanisms. Colored dots represent exRNAs originating from distinct cellular sources, with each color corresponding to a specific CNS cell type (e.g., astrocytes, microglia, and neurons). Dashed-line frames indicate the blood–brain barrier (BBB). Created in BioRender. Lu, K. (2025) https://BioRender.com/rmfun3m.

Extracellular RNAs (exRNAs) comprise a diverse set of RNA species released from cells, detectable in body fluids such as blood, cerebrospinal fluid (CSF), saliva, and urine [1,2,3]. These include small regulatory RNAs—microRNAs (miRNAs) around 22 nucleotides in length—as well as long noncoding RNAs (lncRNAs), circular RNAs (circRNAs), messenger RNAs (mRNAs), and mitochondrial RNAs (mt-RNAs) encoded by mitochondrial genomes [1,2,3]. In extracellular compartments, these RNAs are exposed to abundant RNases yet persist because they are packaged into protective carriers—membrane-bound extracellular vesicles (EVs), which include distinct populations such as exosomes (originating from the endosomal systemre [2] and microvesicles (which bud directly from the plasma membranere [2], RNA-binding protein complexes (notably Ago2), and lipoprotein particles such as high-density lipoprotein (HDL)—which confer biochemical stability and permit systemic transport [3,4,5,6] (see Figure 1).

Importantly, exRNAs are not simply passive degradation products. They reflect regulated intracellular programs and can act as active effectors upon their transfer to recipient cells [12,17]. Mechanistic studies demonstrate selective enrichment of specific RNA cargo into EVs, with subsequent functional transfer to recipient cells: engineered reporter mRNAs packaged into EVs can be translated in recipient cells [12,84], while miRNAs shuttled via EVs can target and repress corresponding mRNAs [12,17]. These capabilities establish two critical principles: exRNAs can report pre-translational transcriptional states, and exRNA transfer can alter gene expression networks in target cells [12,17,84]. These properties have driven intensive investigations of exRNAs as biomarkers and therapeutic vectors across various diseases, including oncology, cardiovascular conditions, and, more recently, neurological disorders (see Table 1 for disease metabolic contexts linked to transcriptional dysregulation).

Understanding exRNA signaling in brain disease necessitates an appreciation of the major cell types within the central nervous system (CNS) and their respective roles. Neurons, which are highly specialized and energy-demanding cells, play an essential role in cognitive and motor function; their dysfunction leads to release of altered RNA signatures that can provide insights into disease states [20,21,22]. Astrocytes support neuronal metabolism, regulate extracellular neurotransmitter concentrations, modulate ionic balance, and influence the integrity of the BBB [85,86]. When activated, astrocytes adapt their secreted RNA repertoires, which can influence both neuronal health and signaling [87,88,89]. Microglia, the resident immune cells in the brain, respond to injury or infection and can release inflammatory RNAs, propagating immune responses throughout the CNS [90,91,92]. Oligodendrocytes, responsible for myelinating axons, provide metabolic support to neurons [93,94,95]; their dysfunction can lead to distinct RNA signatures relevant to demyelination or degeneration [96,97]. The unique transcriptional profiles and stress responses of these diverse cell types imply that changes in the composition of extracellular RNAs can signal which cellular compartments are affected during disease processes (see Table 1).

**Table 1:** **Metabolic abnormalities and representative transcriptional dysregulation in major CNS disorders**. The table summarizes some disease-specific metabolic phenotypes, representative genes/pathways implicated in transcriptional dysregulation, and downstream cellular consequences.

Disease	Metabolic Abnormalities	Representative Gene and Pathway	Cellular Effects of Transcriptional Dysregulation	References
**Alzheimer’s disease (AD)**	Hypometabolism in the hippocampus and posterior cingulate cortex (low FDG-PET uptake); impaired neuronal insulin signaling	PGC-1α, IRS1, IDE, APP, MAPT	Mitochondrial dysfunction, energy deficiency, enhanced Tau phosphorylation	[21,23,24,28,29,40,43,44]
**Parkinson’s disease (PD)**	Reduced oxidative phosphorylation and complex I activity in substantia nigra dopaminergic neurons; altered lipid metabolism in basal ganglia	PINK1, PARKIN, DJ-1, LRRK2	Impaired mitophagy, oxidative stress accumulation, neuronal death	[30,31,39,50,98]
**Amyotrophic lateral sclerosis (ALS)**	Metabolic imbalance in motor cortex and spinal motor neurons; disrupted glucose and lipid utilization within corticospinal tracts	SOD1, TARDBP, FUS, C9orf72	RNA metabolic dysregulation, mitochondrial deformation, stress-induced neuronal death	[34,35,36,48,52,99,100,101]

**Figure 2:**
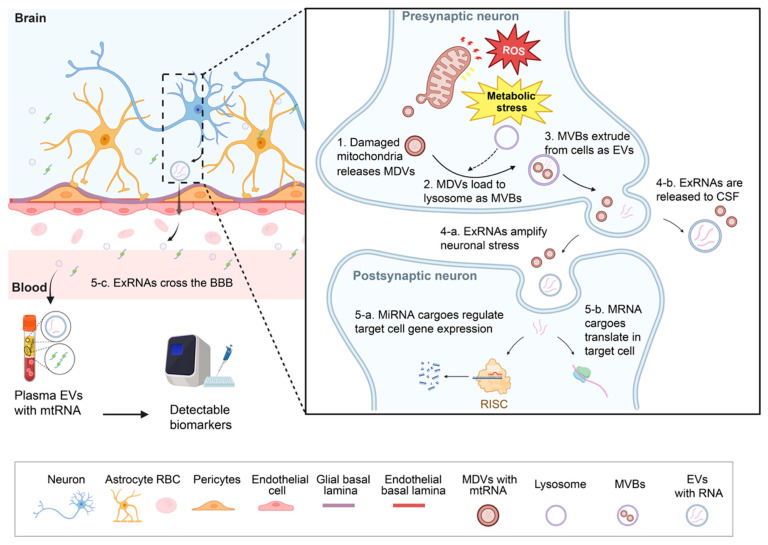
**Oxidative stress–mitochondrial dysfunction coupling drives exRNA remodeling and extracellular vesicle (EV) release**. Neuronal oxidative stress and mitochondrial dysfunction (MD) induce a stepwise pathway that remodels extracellular RNA (exRNA) cargoes [66,67,68,72,83,108,109,110,111]. (1) Damaged mitochondria generate mitochondria-derived vesicles (MDVs) enriched in mitochondrial RNAs (mtRNAs) and oxidatively modified nucleic acids. (2) MDVs are trafficked to lysosomes or incorporated into multivesicular bodies (MVBs). (3) MVBs fuse with the plasma membrane and release their contents as extracellular vesicles (EVs). In this figure, EVs are used as a general term and do not distinguish between exosomes and microvesicles, as both can encapsulate exRNAs. (4) Released exRNAs can amplify neuronal stress or enter cerebrospinal fluid (CSF). (5) A fraction of EV-associated exRNAs crosses the blood–brain barrier (BBB). The BBB is a specialized endothelial interface formed by endothelial cells, pericytes, astrocytic endfeet, and basement membranes, which tightly regulates exchange between the brain and the bloodstream. These processes generate detectable peripheral exRNA signatures in plasma/serum, CSF, or urine that reflect early metabolic and oxidative stress–driven pathology [66,67,68,72,83,108,109,110,111]. Arrows indicate the direction of molecular or vesicular transport. Solid lines represent established biological processes, whereas dashed lines denote specific cellular or structural features highlighted in the schematic. Distinct symbols and different colors of lines are used to distinguish different molecular species, pathways, or cellular components as illustrated. Created in BioRender. Lu, K. (2025) https://BioRender.com/zsaskqg.

Different exRNA classes carry distinct types of biological information. MiRNAs, which are abundant and functionally potent, bind complementary sites in target mRNAs to repress translation or promote degradation [102]. When present extracellularly, they can indicate and modify essential pathways, including those involved in inflammation and mitochondrial homeostasis [12,17]. Specific regulatory miRNAs and noncoding RNAs that are dysregulated in disease tissue—for example miR-7 and miR-153 in PD—are also detectable in extracellular compartments, linking cell type–selective transcriptional changes to peripheral exRNA readouts [12,32,33]. LncRNAs operate through various mechanisms, including scaffolding protein complexes, modulating chromatin, or stabilizing mRNAs [103,104]. Certain disease-relevant lncRNAs, such as BACE1-AS in AD, show significantly elevated levels in plasma-derived small EV (exosome-enriched) fractions, indicating that EV-associated lncRNAs contribute to pathogenic transcript regulation [63]. CircRNAs, characterized by their covalently closed structure, exhibit high stability and may act as miRNA sponges or regulators of transcription [105]. Detection of extracellular mRNAs and mt-RNAs can indicate altered transcriptional programs or responses to mitochondrial stress [72,106,107]; importantly, mRNAs within EVs may be translated in recipient cells, offering a mechanism for direct modification of protein expression in tissues distant from their origin [64,72] (see Figure 2 and Table 1).

The biological context of exRNA carriers influences their interpretation and detection. EVs are a heterogeneous population broadly categorized by their biogenesis: exosomes, which originate from the endosomal system [7,8,9,10], and microvesicles, which bud directly from the plasma membrane [8,9,11]. While exosomes are typically found within the small EV size range (approximately 30–150 nm) [112], they cannot be definitively distinguished from other small EVs by size alone using common isolation methods [113]. Nevertheless, small EV preparations are commonly enriched for exosomes and encapsulate RNA cargo within lipid bilayers, allowing for protection and potential directed delivery [114]. Non-vesicular pools, often associated with RNA-binding proteins like Argonaute, constitute a significant fraction of circulating miRNAs and may reflect both active cellular export as well as passive release from damaged cells [4,5,6]. Additionally, lipoprotein-associated RNAs link metabolic state to RNA transport and can be prevalent in plasma [115,116,117]. The choice of isolation methods—ranging from ultracentrifugation to size exclusion chromatography or immunoaffinity capture—determines the enrichment of these respective pools, impacting biological conclusions drawn from the data [12,13,14,15,16] (see Table 2 for diagnostic and technical considerations).

**Table 2:** Major extracellular RNA (exRNA) carrier classes, their molecular features, biological implications, and methodological considerations. This table summarizes the principal exRNA carrier pools present in blood and other biofluids, highlighting differences in their physical composition, biological interpretability, and technical enrichment biases. Small extracellular vesicles (sEVs)—often enriched for exosomes—encapsulate diverse RNA species within lipid bilayers and reflect regulated endosomal secretion, whereas larger microvesicles originate from plasma-membrane shedding and typically indicate cellular stress or activation. Non-vesicular carriers, including Ago2-associated ribonucleoprotein complexes and lipoprotein-bound RNAs, represent substantial fractions of circulating RNA and may report metabolic state or cell damage rather than directed secretion. Because different isolation approaches (e.g., ultracentrifugation, SEC, immunoaffinity capture) selectively enrich specific carrier types, methodological choices critically shape the downstream interpretation of exRNA profiles. These considerations are essential for evaluating exRNA-based biomarkers and for comparing datasets across studies. SEC, size-exclusion chromatography.

Carrier Type	Composition & Physical Features	Biological Roles/Interpretive Meaning	Isolation Method Bias	Key Considerations	References
**Small EVs (30–150 nm) (commonly enriched for exosomes)**	Lipid bilayer vesicles; contain miRNA, lncRNA, circRNA, mRNA, proteins	Reflect regulated endosomal secretion; can mediate targeted intercellular communication	Ultracentrifugation, density gradients, SEC, immunoaffinity capture	Size cannot define exosomes; population is heterogeneous	[118,119,120]
**Microvesicles (100–1000 nm)**	Shed from the plasma membrane; carry cytosolic RNA and signaling molecules	Indicate cellular stress, inflammation, activation, or injury	Low-/medium-speed centrifugation, SEC, density separation	Highly heterogeneous; cargo varies by cell state	[121,122,123]
**RBP-associated RNAs (e.g., Ago2-miRNA complexes)**	Non-vesicular ribonucleoprotein complexes; major component of circulating miRNAs	Reflect basal RNA export or passive release from damaged cells	Enriched in post-EV supernatants; protein affinity purification	Do not represent EV biology; dominate plasma miRNA pools	[4,5,124,125]
**Lipoprotein-associated RNAs (HDL, LDL)**	RNAs bound to apolipoproteins; abundant in plasma	Link lipid metabolism with RNA transport; prevalent in circulation	Density flotation, precipitation reagents; present in “EV-free” fractions	Strongly influenced by metabolic state; interpret with lipid context	[116,117,126,127,128,129]
**Total mixed exRNA (unfractionated plasma)**	Combination of vesicular and non-vesicular RNAs	Reflects global RNA shifts but lacks cellular specificity	Commercial precipitation kits, low-speed pelleting	Interpretation difficult; usually dominated by non-vesicular RNA	[4,118,125,129]
**Immuno-captured EV subsets (e.g., neuron-derived EVs)**	EVs isolated via cell-type-specific surface markers	Highest interpretability for CNS diseases; enriched in cell-type-specific RNAs	Depends on antibody specificity and epitope exposure	Lower yield; potential nonspecific binding; high specificity	[69,70,71,74]

Mechanistically, cells selectively sort RNAs into carriers through signals recognized by RNA-binding proteins, sequence motifs, or stress-responsive pathways [130,131,132]. Factors such as neuronal activity, synaptic stimulation, oxidative stress, and mitochondrial dysfunction can modulate RNA sorting and release, resulting in dynamic exRNA repertoires that track real-time cellular states [13,14,15,16,66,67]. Once in circulation, clearance mechanisms—such as hepatic, splenic, and renal uptake—impact the half-life and detectability of exRNAs [133]. Various detection methods exist, ranging from bulk sequencing to targeted RT-qPCR or droplet digital PCR (ddPCR) and other quantification platforms, with strategies aimed at enriching for cell type-specific EVs offering improved specificity for CNS exRNA signals [69,70,71] (refer to Figure 1 for detection strategy mapping and Table 3 for platform comparisons).

**Table 3:** Summary of major analytical platforms for extracellular RNA (exRNA) detection and their methodological characteristics. This table compares the principal technologies used to detect circulating exRNAs, including sequencing-based, PCR-based, hybridization-based, and emerging single-EV or biosensor platforms. For each method, key analytical features—such as underlying principles, sensitivity, multiplexing capacity, and suitability for low-abundance targets—are highlighted. Differences in bias, dynamic range, and required input influence the interpretation of EV-associated and non-vesicular RNA signals across biofluids. Together, these comparisons provide a framework for selecting optimal detection strategies based on study aims, sample type, and desired resolution, as well as for contextualizing data derived from diverse exRNA profiling approaches. SERS, surface-enhanced Raman scattering; POCT, point-of-care testing.

Platform	Principle	Main Strengths	Main Limitations	Typical Role in exRNA Studies	References
**Small RNA/total RNA next-generation sequencing (NGS)**	Library prep from total or size-selected RNA followed by high-throughput sequencing	Unbiased, genome-wide profiling; wide dynamic range; detects novel RNAs, splice variants, fusions	Higher cost; longer turnaround; higher input requirement; complex bioinformatics and batch effects	Discovery-phase profiling of EV and non-vesicular exRNA (miRNA, lncRNA, circRNA, mRNA) in plasma/CSF; construction of disease signatures and pathway maps	[134,135,136,137]
**RT-qPCR**	Reverse transcription followed by quantitative PCR with sequence-specific primers/probes	High sensitivity for moderate–high abundance targets; inexpensive; fast; widely available	Requires a priori target selection; relative (not absolute) quantification; normalization difficulties in biofluids; limited multiplexing	Validation of miRNA/lncRNA biomarkers; small targeted panels for longitudinal monitoring	[134]
**Droplet digital PCR (ddPCR)**	Partitioning RT-PCR reactions into thousands of droplets, end-point fluorescence counting for absolute quantification	Very high analytical sensitivity and precision; absolute copy-number detection; ideal for rare exRNAs	Specialized equipment; higher cost per sample; low multiplexing capacity	Precise quantification of rare EV-miRNAs or lncRNAs; low-copy CNS exRNAs in plasma/CSF; EV-mRNA mutation detection	[138,139,140]
**miRNA/mRNA microarrays**	Hybridization of labeled cDNA/cRNA to probe panels; fluorescence-based detection	Medium–high multiplex capacity; relatively inexpensive; simpler workflow than NGS	Limited to known targets; lower sensitivity and narrower dynamic range than NGS and ddPCR; cross-hybridization background	High-throughput screening of predefined miRNA/mRNA panels; large-cohort exRNA biomarker screening	[134]
**NanoString nCounter/barcode hybridization panels**	Probe hybridization with color-coded barcodes, direct digital counting (no amplification)	High reproducibility; medium–high multiplexing; tolerates low or partially degraded input; no PCR bias	Sensitivity lower than NGS and ddPCR; restricted to panel design; platform-specific normalization needed	EV-miRNA or EV-mRNA analysis from low-input EV RNA; cohort screening with targeted panels	[141,142]
**Single-EV/single-particle RNA methods (microfluidic or droplet-based)**	Isolation or barcoding of individual EVs, followed by cDNA synthesis and sequencing or targeted RNA assays	Resolves heterogeneity between EV subpopulations; links RNA cargo to EV size/phenotype	Technically demanding; expensive; low throughput; not standardized	Mechanistic studies of EV cargo selection; exploratory single-EV diagnostics	[143,144,145]
**Biosensor-based exRNA detection (electrochemical, SERS, etc.)**	Surface-immobilized probes capture specific RNAs; electrical or Raman-based signal detection	Ultrafast detection; tiny sample requirement; potential for point-of-care devices	Limited to few targets; lacks broad clinical validation; mainly proof-of-concept	On-chip detection of selected EV miRNAs or tumor exRNAs; potential POCT applications	[146,147,148,149,150]

**Figure 3:**
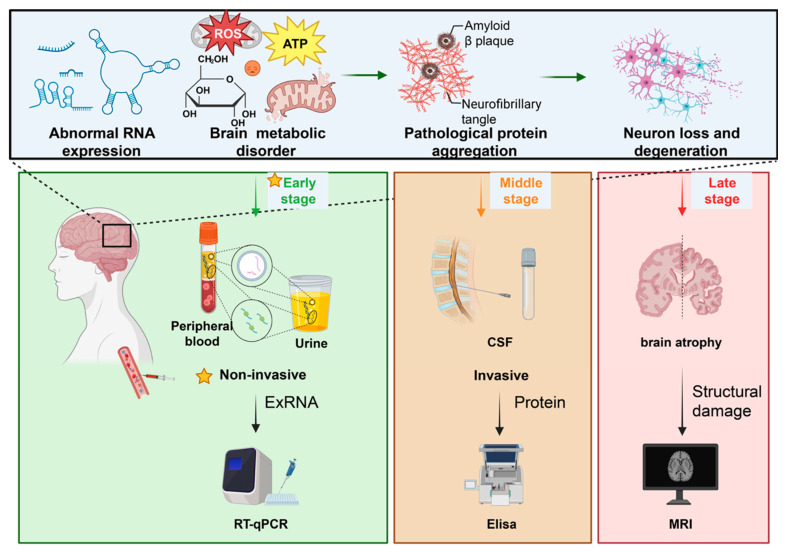
**Timeline of biomarker windows in neurodegeneration**. Schematic timeline showing the sequential emergence of molecular, biochemical, and structural alterations during neurodegenerative disease progression. Early-stage disturbances include abnormal gene expression, mitochondrial dysfunction, oxidative stress (reactive oxygen species, ROS), and impaired energy metabolism (adenosine triphosphate, ATP), which together generate extracellular RNA (exRNA) signatures detectable in peripheral biofluids [61,62,63,64,65,66,67,72,83,151,152,153]. These minimally invasive exRNA windows—accessible through plasma, serum, urine, or saliva—precede conventional biomarker changes [61,62,63,64,65]. Mid-stage changes feature the appearance of proteinopathy markers such as amyloid-β, phosphorylated tau, and α-synuclein, typically measured in cerebrospinal fluid (CSF) [43,48,49,50,51,52]. Late-stage biomarkers reflect overt neurodegeneration and brain atrophy, detectable by structural neuroimaging (magnetic resonance imaging, MRI) [21]. Overall, exRNAs provide earlier molecular readouts than traditional protein or imaging biomarkers. Solid arrows indicate the direction of disease progression, molecular changes, or diagnostic workflows. Dashed lines represent stage associations or conceptual links rather than direct physical processes, and dashed frames highlight specific diagnostic windows or regions of interest. Different symbols denote sampling or methodological features, with the star indicating non-invasive approaches. Distinct background colors indicate disease stages and corresponding diagnostic strategies: green, early-stage non-invasive exRNA-based detection; orange, mid-stage invasive protein-based measurements (e.g., CSF); red, late-stage structural damage assessed by imaging modalities. Created in BioRender. Lu, K. (2025) https://BioRender.com/3nfw9th.

Together, these biological and technical considerations explain why exRNAs present a minimally invasive window into early transcriptional and metabolic abnormalities within the brain. Their selective packaging and stress-dependent release suggest that exRNA profiles may precede downstream protein aggregation and structural damage (see Figure 3), positioning them as promising candidates for earlier diagnosis, prognosis, and mechanistic readouts. However, to realize their full potential as biomarkers and therapeutic targets, it is critical to rigorously address variables related to carrier biology, cellular origin, and methodological confounding factors.

## 3. Extracellular RNA Trafficking and the Blood–Brain Barrier

For exRNAs to serve as peripheral biomarkers of brain state or to mediate brain–periphery signaling, they must reach the circulation or other accessible fluids. The BBB is a specialized endothelial interface that tightly controls exchange between the CNS and the blood; it restricts passive diffusion of large or hydrophilic molecules while permitting regulated transport of nutrients, hormones, and selected vesicles [80,81,82]. Multiple complementary routes allow CNS-derived exRNAs or their carriers to appear in peripheral fluids: direct release into CSF with subsequent drainage to the blood, transcytosis across the BBB, and trafficking via meningeal or perivascular pathways that connect brain interstitium to lymphatic and systemic circulation [53,54,55,56,57] (see Figure 2).

CSF as an intermediary: neurons and glia release EVs and non-vesicular exRNAs into the interstitial fluid and CSF [154,155,156]. CSF circulates through ventricular and subarachnoid spaces and drains to peripheral lymphatics and venous sinuses [157,158]; as a result, CSF-derived exRNAs can enter the blood, especially at interfaces such as the cribriform plate, meningeal lymphatics, and arachnoid granulations [53,54,55]. While CSF sampling (lumbar puncture) remains more invasive than blood draws, direct CSF analysis can provide high CNS specificity and has been valuable for identifying brain-derived exRNA candidates [155,159,160] (see Table 4).

**Table 4:** **Current diagnostic paradigms and limitations of major CNS disorders**. The table lists common neuroimaging and fluid biomarkers used clinically, the typical disease stage at which these indicators become abnormal, and their limitations with respect to detecting early transcriptional or metabolic alterations. This table highlights the gap that exRNA-based assays may fill by reporting earlier molecular dysfunction. Arrows indicate the direction of change in biomarker levels (↑, increase; ↓, decrease).

Disease	Neuroimaging Diagnostic Indicators	CSF Biomarkers	Diagnostic Stage	Limitations	References
**Alzheimer’s disease (AD)**	FDG-PET hypometabolism, MRI-detected cortical atrophy	Aβ42 ↓/Tau ↑	Mostly diagnosed at middle to late stages	Cannot reflect early transcriptional or metabolic alterations	[43,161]
**Parkinson’s disease (PD)**	DAT-SPECT imaging	α-synuclein oligomers, DJ-1	Diagnosed after symptom onset	Lacks indicators of early neuronal metabolic abnormalities	[49,50,51]
**Amyotrophic lateral sclerosis (ALS)**	MRI-detected cortical atrophy, electromyography (EMG)	NfL	At the stage of neuronal injury	Unable to monitor early metabolic imbalance or RNA regulatory dysfunction	[48,52]

Vesicular transcytosis and endothelial transport: Brain endothelial cells can internalize EVs from the abluminal (brain) side and transcytose them to the luminal (blood) side, or vice versa, enabling bidirectional vesicular trafficking across the BBB [53,162,163]. Experimental tracer studies in rodents, including using radiotracers, fluorescent/luciferase-tagged EVs, and BBB-on-chip live imaging to directly visualize transcytosis, show that peripheral small EVs cross the BBB and distribute within brain parenchyma and vasculature, and conversely, brain-derived EVs can be detected in the circulation after crossing endothelial barriers [53,162,163]. Clathrin- and caveolin-mediated transcytosis are cellular routes for moving material across endothelial cells: clathrin forms a protein coat that helps internalize cargo into vesicles, while caveolin stabilizes small membrane invaginations (caveolae) that ferry material across the cell [164,165,166]. The efficiency of these pathways depends on endothelial “activation”—whether the cells are quiescent or reactive—with inflammatory signals, oxidative stress, or aging increasing endocytic activity and vesicle formation [73,164,167,168,169,170]. Consequently, inflammation and vascular aging common in neurodegenerative disease can raise vesicular trafficking across the BBB and change how much CNS-derived exRNA reaches the blood.

Perivascular, glymphatic, and meningeal routes: The glymphatic system and meningeal lymphatic vessels provide conduits for interstitial fluid and solute clearance from brain tissue to peripheral lymph nodes and blood [171,172,173,174,175]. Sleep, arterial pulsatility, and aquaporin-4–mediated astrocytic water flux influence glymphatic clearance [171,173,175,176,177], and impairment of these pathways in aging or disease can alter exRNA drainage patterns [173]. Such routes enable EVs and non-vesicular RNAs from parenchymal cells to reach dural lymphatics and systemic circulation without direct endothelial transcytosis, giving an alternate explanation for how brain signals become detectable peripherally [171,178].

Cellular sources and markers for CNS origin: Assigning peripheral exRNAs to a CNS source requires orthogonal evidence. Neuron-derived EVs (NDEs) can be enriched from plasma using immunocapture against neuronal surface proteins (for example L1CAM/SNAP25/MAP2-related markers), enabling detection of neuron-enriched transcripts and proteins [69,70,71,74]. Astrocyte- or microglia-derived EVs have likewise been isolated using cell-type–specific surface antigens [74,75,76], although antibody specificity and the extent of marker shedding remain technical constraints. Parallel approaches combine CSF–blood comparisons, tissue correlation, and single-cell or spatial transcriptomics to triangulate origin [179,180]. Importantly, many reported peripheral exRNA signals may be composite—reflecting CNS release, peripheral responses to CNS pathology, or systemic metabolic dysregulation—so careful experimental design is required to interpret provenance. 

Functional uptake by peripheral tissues and immune cells: Once in the circulation, exRNA-containing EVs can interact with peripheral immune cells, endothelial cells, liver, and kidney, potentially altering systemic responses and generating feedback to the brain [77,78,79]. Studies show preferential uptake of peripheral sEVs by innate immune cells including monocytes and macrophages [77,78,79]. When peripheral EVs cross into the CNS they are often taken up by microglia and astrocytes, and aged donor EVs elicit distinct glial activation patterns that include changes in GFAP and CD68 expression and selective cytokine responses [181]. These interactions complicate biomarker interpretation but also reveal pathways by which brain pathology can influence whole-organism physiology.

Influence of disease state on trafficking efficiency: Neuroinflammation, aging, vascular dysfunction, and BBB breakdown—common features of neurodegenerative disorders—alter barrier permeability and transcytotic capacity, thereby modifying the quantity and composition of CNS-derived exRNAs reaching the periphery [53,54,55,56,57,80,81,82]. For example, inflammatory cytokines can upregulate endothelial vesicle transport and facilitate immune cell infiltration [182,183] while oxidative stress and microvascular pathology can increase paracellular leak [184,185]. For biomarker studies this implies that elevated peripheral exRNA levels may indicate increased CNS release, enhanced barrier permeability, altered clearance, or increased peripheral production [186,187]; disentangling these possibilities requires integrated measures such as CSF sampling, multimodal neuroimaging (MRI, Positron Emission Tomography (PET), or related techniques), and cell type-specific enrichment [186,187] (see Table 4).

From a technical standpoint, trafficking complexity mandates deliberate sample selection and rigorous controls. CSF provides high CNS specificity but limited scalability; plasma and serum enable large-scale and longitudinal sampling but require strategies to enrich for CNS-derived material (immunocapture, neuronal marker selection) or to integrate orthogonal measures (imaging, CSF biomarkers) that support CNS origin. Standardized timing of sampling relative to circadian, sleep, or treatment variables is important given glymphatic and activity-dependent influences on exRNA release [175,188,189,190]. Finally, combining carrier-specific isolation (EV vs. protein-bound) with cell-type enrichment and sequencing can improve both sensitivity and interpretability (Figure 1, Figure 2 and Figure 3, Table 2, Table 3 and Table 4).

In summary, multiple anatomical and cellular pathways enable CNS-derived exRNAs to reach peripheral fluids, and disease states modulate these routes. Understanding trafficking mechanisms and applying rigorous capture strategies are essential to distinguish true brain-derived signals from peripheral noise and to exploit exRNAs reliably as biomarkers or effectors in neurodegenerative disease.

## 4. Functional Roles of Extracellular RNAs in Neural Physiology

exRNAs participate in regulated intercellular communication within the brain and in bidirectional brain–periphery signaling [191,192,193,194]. Neuronal activity, particularly at excitatory glutamatergic synapses, drives calcium-dependent secretion not only of neurotransmitters but also of membrane-bound extracellular vesicles and associated RNA cargo [192]; stimulated cortical neurons secrete EV populations with selectively enriched RNA repertoires that can be preferentially taken up by other neurons, indicating an activity-dependent route of interneuronal messaging distinct from classical synaptic transmission [191,195] (see Figure 2). This regulated release enables exRNA repertoires to report ongoing neuronal programs—plasticity, metabolic demand, and stress responses—and provides a mechanism for rapid, context-dependent transfer of molecular information [191,192,193,194].

After uptake, exRNAs can exert functional effects in recipient cells. EV-packaged mRNAs have been translated into detectable proteins in recipient cells, and transferred miRNAs repress target mRNAs, thereby reshaping gene expression networks [12,17]. These observations show that exRNAs can modify protein synthesis and cellular phenotype in non-cell-autonomous ways [96,196,197,198,199,200]: in neurons this can manifest as altered synaptic protein composition, receptor expression, or local metabolic enzyme abundance [96,196,197,198,199,200]; in glia it can change inflammatory tone, metabolic support, or BBB-regulating functions [200]. The ability of exRNA to influence translation and post-transcriptional control provides a mechanistic link between cellular state in one compartment and functional change in another.

Glia–neuron exRNA exchange is particularly important for circuit homeostasis. Astrocytes secrete RNAs that regulate neuronal excitability and metabolism, and microglia release RNA-containing vesicles that modulate synaptic pruning and immune signaling [198,201,202,203]. Preferential uptake patterns have been observed: peripheral small EVs entering the brain are often internalized by microglia and astrocytes, whereas neuronal uptake depends more on specific surface interactions [181,198,204]. Because astrocytes and microglia shape extracellular environment, synaptic support, and immune surveillance, exRNA exchange among these cell classes contributes to fine-tuning of network function and adaptive responses to activity or stress [198,201,202,203].

Beyond local exchanges, exRNAs mediate brain–periphery crosstalk [178,205,206]. EVs and non-vesicular RNAs released from brain cells can drain via CSF or traverse the BBB to reach peripheral immune cells, liver, and kidney, where their cargo can reprogram immune responses, endothelial function, or metabolic pathways [53,54,55,56,57,181]. Conversely, peripheral cells—immune cells, endothelial cells, adipose, liver, and muscle—secrete vesicles that can cross into the CNS and modulate glial reactivity or neuronal function, establishing a bidirectional axis that integrates neural state with whole-body physiology [202,205,207] (Table 5).

**Table 5:** **Extracellular vesicle-derived microRNAs (EV-miRNAs) mediating systemic organ–brain communication**. The table lists representative EV-miRNAs associated with different organ–brain axes, summarizes associated CNS effects and disease contexts, and notes potential biomarker or therapeutic relevance. Abbreviations: CNS, central nervous system; AD, Alzheimer’s disease; PD, Parkinson’s disease; ASD, autism spectrum disorder; TBI, traumatic brain injury; MI, myocardial infarction; MSC, mesenchymal stem cell; BDNF, brain-derived neurotrophic factor; CKD, chronic kidney disease; MS, multiple sclerosis; EV, extracellular vesicle.

Axis	Representative EV-miRNAs	Associated CNS Effects/Diseases	Potential Biomarker or Therapeutic Relevance	Validation	References
**Gut–Brain**	miR-146a, miR-206-3p, miR-155-5p	Dysbiosis-linked neuroinflammation; anxiety, depression, AD, PD, ASD	Candidate biomarkers for gut–brain disorders; therapeutic modulation of dysregulated miRNAs	Observational/correlative; limited mechanistic linkage for specific miRNAs	[208,209,210,211,212]
**Lung-Brain**	miR-21, miR-145, miR-217, miR-374a-5p	Lung cancer-derived EVs promote brain metastasis; TBI-associated inflammation	Circulating EV-miRNAs as markers for metastasis risk; possible targets to block BBB disruption	In vivo model support (causal evidence for EV-miRNA in brain metastasis); plus human biomarker validation in cohorts	[213,214,215,216]
**Heart-Brain**	miR-1, miR-27a, miR-29b, miR-340, miR-424, miR-17-92 cluster	Post-MI EV-miR-1 induces hippocampal microtubule damage; EV-miR-27a linked to oxidative stress in heart failure; overlapping EV-miRNAs in stroke and MI	Diagnostic candidates for cardiogenic dementia and stroke; therapeutic miRNA delivery (e.g., MSC-EVs)	In vivo model support (exosome-mediated heart-to-brain transfer demonstrated)	[217,218,219,220,221,222,223,224,225]
**Muscle-Brain**	MyomiRs (miR-1, miR-133a, miR-206, miR-499, miR-486, miR-29b-3p)	Exercise-induced neuroprotection; traumatic brain injury (TBI); regulation of BDNF, neuroplasticity, neuropathic pain	Serum exosomal myomiRs as biomarkers for neurodegeneration and TBI prognosis	Observational/correlative (circulating/exosomal myomiRs); mechanistic CNS effects remain limited/indirect	[226,227,228]
**Adipose-Brain**	miR-155, miR-21, miR-425, miR-29a, miR-9-3p, miR-33	Obesity/diabetes-induced neuroinflammation; cognitive decline; altered hypothalamic signaling	Adipose-derived EV-miRNAs as liquid biopsy markers for obesity-associated cognitive impairment	In vivo model support (adipose EVs/miRNA cargo modulate brain outcomes in obesity/metabolic disease models)	[229,230,231,232]
**Kidney-Brain**	miR-29a, miR-223, miR-27a, miR-326, miR-34a, miR-17, miR-126	Chronic kidney disease (CKD) linked to cognitive impairment; ischemic brain injury; EV-miR-34a shared between brain and kidney	Novel liquid biopsy markers to monitor CNS complications in CKD	Observational/correlative (CKD biofluid EV-miRNAs as biomarkers); limited direct kidney-to-brain causal EV-miRNA evidence	[233,234,235,236,237]
**Immune-Brain**	miR-146a, miR-155, miR-124, miR-21-5p, miR-409-3p	Neuroinflammation in AD, PD, MS, TBI; microglial activation or polarization	Targets for EV-based immunomodulation (e.g., engineered EV-miR-124 delivery for neuroprotection)	In vivo model support (EV-miRNA therapy/functional modulation of neuroinflammation and recovery)	[210,238,239,240,241,242]

In healthy conditions, these exchanges support plasticity, metabolic coordination, and immune surveillance [202,243,244]. Activity-dependent control and metabolic responsiveness ensure that secreted RNA repertoires convey context-dependent information [199]; this dynamic regulation underlies the sensitivity of exRNA profiles to early shifts in neuronal function and cellular stress before irreversible structural injury occurs [115,199,200]. Thus, the physiological roles of exRNAs provide both the rationale for their use as early biomarkers and the mechanistic basis by which maladaptive exRNA signaling can drive pathology in neurodegenerative disease [115,199,200] (see Figure 1, Figure 2 and Figure 3).

## 5. Altered exRNA Signaling in Neurodegeneration

In neurodegenerative and related brain disorders, regulated exRNA release becomes perturbed and can contribute directly to pathogenesis [245,246,247,248]. A convergent early axis across AD, PD, and ALS is mitochondrial dysfunction and oxidative stress, which promote altered RNA sorting and increased release of mt-RNAs and oxidatively modified nucleic acids via mitochondria-derived vesicles (MDVs) and multivesicular bodies [83,108,109,110]. These mt-RNAs and oxidized RNAs behave as damage-associated molecular patterns (DAMPs) that engage innate immune sensors (for example cGAS–STING and Toll-like receptors), amplify neuroinflammation, and create feed-forward loops linking metabolic injury to immune activation [108,109].

Inflammatory glia and stressed neurons secrete vesicles enriched for specific microRNAs and long noncoding RNAs that elicit maladaptive responses in recipient cells [68,154,249,250]. For example, in neuroinflammatory environments, activated microglia and infiltrating macrophages release EVs enriched in miR-21; this exRNA cargo can trigger Toll-like receptor-7 (TLR7)–dependent necroptosis in neighboring neurons [17]. Similarly, glia-derived EV delivery of miR-146a-5p downregulates synaptic proteins such as synaptotagmin-1 and neuroligin-1, reducing dendritic spine density and impairing excitatory transmission [68]. In ALS, mislocalization of RNA-binding proteins such as TDP-43 and disrupted nucleocytoplasmic transport plausibly alter exRNA export and composition, linking core RNA-metabolism defects to extracellular signaling and propagation of pathology [38,46,47]. exRNAs also interact with proteinopathy propagation: EVs can carry pathogenic proteins such as tau or α synuclein alongside RNA cargo, and these mixed cargos may co-operate to promote spread of misfolded proteins across brain regions [4,5,251]. Although numerous studies describe altered exRNA release during neuroinflammation, causal investigations of the molecular machinery that controls exRNA sorting in CNS cells remain limited. Recent work has begun to provide mechanistic insights; for example, YB-1 in microglia was shown to regulate selective loading of miR-223 into extracellular vesicles [252], and genetic manipulation of YB-1 altered EV cargo composition, neuroinflammatory responses, and cognitive outcomes in an AD model [252]. Aside from such emerging examples, targeted perturbation studies—particularly those using CRISPR-based disruption of RNA-binding proteins—are still scarce, underscoring a major knowledge gap in understanding how exRNA export pathways mechanistically contribute to CNS pathology.

exRNAs also intersect with propagation of proteopathic species. Extracellular vesicles can carry pathogenic proteins (for example tau or α-synuclein) alongside RNA cargo, and these mixed cargos may cooperate to promote spread of misfolded proteins across brain regions [4,5,251]. RNA species within EVs can modulate recipient cell proteostasis and inflammatory pathways, potentially facilitating seeding or reducing clearance of aggregated proteins.

Peripheral contributions and systemic coupling further complicate interpretation. CNS pathology alters peripheral metabolism and immune tone via hypothalamic–pituitary–adrenal axis activation, autonomic outputs, and cytokine signaling, provoking peripheral cell responses that change circulating exRNA pools [253,254,255,256,257]. Conversely, peripheral EVs derived from aged immune cells, adipose, liver, or gut tissues can cross into the brain and exacerbate glial reactivity or synaptic dysfunction [181]. Thus, patient plasma exRNA signatures often represent a composite of central release, peripheral responses to CNS injury, and systemic metabolic dysregulation [181,253,254,255,256,257].

Oxidatively modified nucleotides and elevated mt-RNA species detectable in plasma or EVs provide mechanistic readouts of early cellular stress. For example, increased abundance of mitochondrial transcripts (MT-ND, MT-CO family members) in plasma EVs and higher levels of RNA oxidation markers such as 8-hydroxyguanosine (8-OHG) and 8-oxo-guanosine (8-oxoGuo) have been reported in preclinical models and human samples [72,83], supporting the concept that exRNA patterns encode mitochondrial health and oxidative damage prior to extensive neurodegeneration.

Collectively, these pathological mechanisms indicate that exRNAs are not mere biomarkers but can act as active agents that propagate inflammation, impair synaptic integrity, and contribute to spread of proteopathic species. This dual role heightens both diagnostic opportunities and therapeutic challenges: interventions that neutralize maladaptive exRNA signaling could slow progression, but require detailed mapping of cellular sources, cargo identity, and recipient pathways to avoid unintended consequences (see Figure 1, Figure 2 and Figure 3 and Table 1, Table 4 and Table 5 for schematic summaries and representative molecular examples).

## 6. Extracellular RNAs as Biomarkers: Technical and Conceptual Considerations

exRNAs offer promising biomarker information, but translating exRNA signals into robust clinical assays requires careful attention to biological context and methodological detail. Key considerations span sample selection and handling, isolation and profiling strategies, normalization and quantification, and study design for discovery and validation.

Sample type and pre-analytical handling: choice of biofluid (plasma, serum, CSF, saliva, urine) determines CNS specificity, invasiveness, and scalability. Cerebrospinal fluid provides the highest likelihood of CNS origin but requires lumbar puncture [258,259], whereas blood is readily obtainable for longitudinal screening but contains large non-CNS RNA pools [58,59,60] (see Table 2 for different types of exRNA). Pre-analytical variables—anticoagulant type, time to processing, centrifugation steps, freeze–thaw cycles, and storage temperature—substantially affect measured exRNA levels and the relative representation of vesicular versus protein-bound pools [260,261,262,263,264]; standardization of these steps is essential for reproducibility [260,261,262,263,264].

Isolation and enrichment approaches: methodological choice strongly shapes which RNA compartments are assayed. Total plasma/serum profiling captures both vesicular and non-vesicular RNAs [265]; EV enrichment methods (ultracentrifugation, size-exclusion chromatography, precipitation kits, immunoaffinity capture) bias toward membrane-enclosed cargo but differ in purity and yield [266,267]. Immunocapture using cell-type markers (for example neuron-derived EV markers using L1CAM or other neuronal antigens) can increase CNS specificity [69,70,71] but depends on antibody specificity and marker retention. Non-vesicular isolates (Ago1-bound RNAs or lipoprotein-associated RNAs) require distinct extraction protocols [4,5,116,117,268,269]. Reporting the isolation method and characterizing preparations (particle size, protein markers) are critical for interpretation [116,268,269].

Profiling technologies and sensitivity: next-generation sequencing (small-RNA or total RNA sequencing) enables discovery but requires bioinformatic care for low-input samples and for distinguishing fragmented RNAs from biologically relevant species [270]. Targeted assays (RT-qPCR, droplet digital PCR) provide high sensitivity and absolute quantification for selected candidates (useful for validation and clinical translation) and can detect low-abundance RNAs such as PHGDH or mt-RNAs [64,65]. Choice of platform should balance discovery breadth, quantitative accuracy, cost, and clinical feasibility. A detailed comparison of platform principles, strengths, and limitations is provided in Table 3.

Normalization and data analysis: proper normalization is a major challenge because classical intracellular reference genes are not applicable to extracellular compartments. Strategies include spike-in controls, global mean normalization, use of stable endogenous small RNAs identified per cohort, and normalization to particle or protein content for EV studies [271]. Statistical models must account for batch effects, confounders (age, sex, comorbidities, medication), and compositional differences between vesicular and non-vesicular fractions [271,272,273]. Cross-study comparisons require transparent reporting of normalization strategies.

Specificity, cellular origin, and biological interpretation: peripheral exRNA profiles reflect a mixture of CNS release, peripheral responses to CNS pathology, and unrelated systemic signals [271]. Demonstrating CNS origin ideally combines multiple lines of evidence: elevated signal in CSF, enrichment in neuron-derived EV fractions, correlation with brain tissue expression, or temporal association with CNS pathology (imaging, CSF protein biomarkers). Immunocapture of cell-type specific EVs (neuronal, astrocytic, microglial) and orthogonal tissue comparisons strengthen causal relationships [69,70,71], but remain imperfect due to marker shedding and antibody limitations.

Study design, cohorts, and validation: many published exRNA biomarker studies are small, cross-sectional, or lack external replication. Robust biomarker development requires adequately powered discovery cohorts, independent validation sets, longitudinal sampling to assess temporal dynamics and predictive value (conversion from MCI to AD, progression in ALS), and inclusion of diverse populations [274,275]. Multimodal integration—combining exRNA panels with neuroimaging (MRI, PET), CSF protein biomarkers, metabolomics, and clinical measures—can increase diagnostic accuracy and provide mechanistic context [61,62,151]. Reporting standards (sample metadata, isolation methods, sequencing depth, data processing) are essential to enable meta-analyses [273].

Analytical sensitivity versus biological variability: highly sensitive assays can detect low-abundance exRNAs but risk amplifying biologically irrelevant noise or contamination [268,276,277,278]. Conversely, biological variability—circadian rhythms, recent activity, diet, comorbid conditions, or acute illness—can confound signals [276]. Controlled sampling (fasting state, time of day), paired CSF–blood sampling when feasible, replication across independent cohorts, and orthogonal validation (different platforms, spike-ins, synthetic standards) help distinguish true biological signals from noise.

From discovery to clinical assay: for clinical translation, candidate exRNAs must demonstrate analytical validity (repeatability, robustness), clinical validity (sensitivity, specificity, prognostic value) and clinical utility (improvement in decision-making or outcomes) [279,280]. Multiplex panels or machine-learning classifiers combining exRNA features with established biomarkers (imaging, CSF proteins) may offer the best path to actionable tests [271,281,282,283,284], but require prospective validation in real-world settings.

In summary, exRNA biomarker development is promising but technically demanding [271]. Adoption of standardized pre-analytical (SOPs), transparent reporting of isolation and profiling methods, careful normalization, orthogonal mapping of cellular origin, multimodal integration, and rigorous longitudinal validation are prerequisites to translate exRNA signatures into reliable clinical tools.

## 7. Disease-Specific Evidence

In many brain diseases, mitochondrial damage or mitochondria dysfunction (MD) and oxidative stress are early and often preclinical events [39,40,41,285] (Table 1). Damaged mitochondria can transfer mitochondrial nucleic acids into EVs via mitochondria-derived vesicles (MDVs) [109,110]. The released mitochondrial nucleic acids act as damage-associated molecular patterns (DAMPs) that activate cGAS-STING/TLR signaling [108,109,110], establishing a biological cascade characterized by mitochondrial injury-driven exRNA release and inflammatory activation [109], thereby providing mechanistic support for the elevation of exRNAs in peripheral fluids.

### 7.1. Alzheimer’s Disease and Mild Cognitive Impairment

AD exhibits hippocampal and cortical neuronal loss and synaptic density reduction; impaired brain glucose utilization, mitochondrial dysfunction, and transcriptomic/splicing abnormalities are evident in affected regions (Table 1). Current clinical testing emphasizes CSF amyloid-β42 and tau or imaging of amyloid deposition [43,161,286], markers that generally reflect downstream protein aggregation and structural injury [43]. However, impaired energy metabolism and oxidative stress often precede classical fluid biomarkers and plaque accumulation in AD and its prodrome, MCI [41,42,43]. By contrast, exRNAs capture an earlier layer of transcriptional and metabolic disturbance: circulating and EV-associated miRNA panels distinguish MCI/AD from controls and relate to amyloid/tau pathology [61,62], exosomal long noncoding RNAs such as BACE1-AS correlate with amyloid processing and enhance diagnostic performance when combined with MRI measures (entorhinal volume, cortical thickness) [63], and plasma EV mRNA profiling has identified transcripts including PHGDH and multiple mitochondrial genes that may appear years before symptom onset [64,65] (Figure 1, Figure 2 and Figure 3; Table 1 and Table 2). Oxidatively modified RNAs and elevated mitochondrial transcripts (for example NADH dehydrogenase subunits *MT-ND1*, *MT-ND2*, *MT-ND3*, *MT-ND4*, *MT-ND4L*, *MT-ND5*, *MT-ND6* mRNA, ATP synthase subunits *MT-ATP6*, *MT-ATP8* mRNA and cytochrome c oxidase subunits *MT-CO1*, *MT-CO2*, *MT-CO3*) in plasma EVs, and markers of RNA oxidation such as 8-hydroxyguanosine (8-OHG), have been reported in preclinical models and human samples [66,72], supporting the view that exRNA patterns capture early metabolic and transcriptional disturbance in AD.

Quantitative performance metrics illustrate clinical potential in AD. The AUC value (area under the curve) reflects the probability that a biomarker correctly distinguishes between patients and controls, with a value of 1.0 representing optimal classification. Plasma miR-145-5p is negatively associated with CSF Aβ1-42 and is elevated in AD/MCI, achieving an AUC up to 0.77 among tested miRNAs [61]. Multimodal combinations (miRNAs plus cognitive testing or MRI) markedly improve classification; in one but smaller internal validation set certain combinations yielded an AUC of 1.0 [61], and replication in a larger external RT-qPCR cohort confirmed diagnostic value with lower but supportive AUCs [61]. Broader EV-miRNA studies show reproducible signatures across independent cohorts [62]. Community-based screening found plasma exosomal miR-483-5p and miR-502-5p elevated in elderly individuals with MCI, with miR-483-5p reaching AUC 0.901 (79.2% sensitivity, 100% specificity) and miR-502-5p AUC 0.872 (79.2% sensitivity, 83.3% specificity) [151]. Also, when exosomal BACE1-AS is combined with MRI imaging, diagnostic performance is also improved, achieving an AUC of 0.82 [63]. These examples illustrate that individual exRNA candidates and multimodal panels can complement established diagnostics and support feasible, minimally invasive screening strategies for early cognitive impairment.

### 7.2. Parkinson’s Disease

PD is characterized by progressive loss of dopaminergic neurons in the substantia nigra pars compacta [30,31], altered neuronal energy and lipid metabolism with metabolites such as lactate [30,31], and dysregulation of miRNAs that have been linked to dopaminergic neuron vulnerability, including miR-7 and miR-153 [30,31,32,33] (Table 1). In PD, fluid biomarkers have primarily focused on oligomeric α-synuclein and DJ-1 [49,50,51], reflecting downstream synaptic protein aggregation and stress responses [49,50]. In contrast, mitochondrial complex I deficiency, oxidative stress, and metabolic disruption are considered upstream events in the pathological cascade [98], often initiated before the onset of motor symptoms or clear abnormalities in these traditional protein markers. ExRNA studies have detected miRNAs connected to mitochondrial dysfunction and oxidative stress in PD [66,67]. For instance, serum EV miR-137 is elevated in PD and targets antioxidant regulators such as OXR1, providing a mechanistic link between exRNA changes and neuronal oxidative injury [67]. Rotenone and other mitochondrial toxins alter CSF and serum EV-miRNA repertoires in animal models, and overlapping miRNA changes appear in human PD cohorts [66]. CSF signatures generally provide higher CNS specificity, but peripheral blood panels offer greater accessibility for longitudinal screening. PD exRNA work highlights both disease-relevant pathways and the challenge of distinguishing central versus peripheral sources.

### 7.3. Amyotrophic Lateral Sclerosis

ALS is marked by progressive loss of upper and lower motor neurons [99]. In ALS, the most robust fluid biomarkers are NfL and pNfH [48,52], which reflect axonal injury and neurodegeneration [52]. Still, MD and oxidative stress in motor neurons are regarded as earlier pathogenic drivers [287] (Table 1), suggesting that exRNAs associated with these metabolic axes may report molecular perturbations preceding NfL/pNfH elevation. ExRNA research has identified circulating miRNAs with diagnostic and prognostic value [152,153]. Panels including miR-214 and others differentiate ALS patients from controls and correlate with disease progression and survival [152,153]. Mechanistically, ALS is characterized by RNA-binding protein mislocalization (e.g., TDP-43) and disturbed nucleocytoplasmic transport [38,46], processes that plausibly alter exRNA export and composition [38,46,47]. EV-mRNA and miRNA changes reflecting mitochondrial stress and impaired proteostasis have been observed [288], suggesting exRNA profiles may inform both diagnosis and rate of progression [289].

### 7.4. Frontotemporal Dementia and Huntington’s Disease

Evidence for exRNA alterations in FTD and HD is less extensive but growing. Shared molecular themes—transcriptional dysregulation, RNA processing defects, and mitochondrial impairment—predict overlapping exRNA signatures with AD and ALS [16,100,290,291]. Beyond these shared pathways, preliminary biofluid studies have begun to identify disorder-associated exRNA species. In FTD, plasma profiling has reported downregulation of neuron-related miRNAs such as miR-663a, miR-502-3p, and miR-206, which differentiate patients from healthy controls and reflect cortical vulnerability [292]. In HD, circulating miRNAs including miR-10b-5p and miR-486-5p are elevated and correlate with clinical stage [293], suggesting that exRNA signatures mirror progressive striatal degeneration. Nevertheless, larger, replicated cohorts are needed to establish robust biomarkers and to disentangle disease-specific from shared neurodegenerative signals. 

Although several miRNAs, including miR-206, miR-663a, miR-502-3p and miR-10b-5p, have been identified through clinical biomarker studies as differentially expressed in FTD and HD biofluids [292,293,294], their functional roles in neurodegeneration and within CNS-resident cell types remain incompletely understood. Notably, only a limited subset of these miRNAs has been examined in neural contexts. For example, miR-206 has been shown to regulate brain-derived neurotrophic factor (BDNF) availability and synaptic or neuronal resilience in CNS-related disease models [295], while miR-10b-5p has been linked to neuronal transcriptional networks and disease severity in HD brain tissue [294]. In contrast, direct functional interrogation of these and related miRNAs in CNS neurons or glial cells remains scarce. This gap underscores the need to move beyond correlative biomarker discovery toward mechanistic studies that establish causal roles for exRNAs in neurodegenerative pathology.

### 7.5. Multiple Sclerosis and Traumatic Brain Injury

In MS, exRNA changes reflect immune activation and demyelination: circulating miRNAs and EV-associated RNAs correlate with relapse activity, lesion burden, and treatment response [296,297,298]. Among these, miR-155 is consistently elevated in patient biofluids and EV fractions and is associated with Th1/Th17 polarization (inflammatory CD4^+^ T helper cell subsets) and heightened neuroinflammatory activity [299,300]. In contrast, miR-124, a neuron- and microglia-enriched miRNA, is reduced and has been shown to restrain macrophage and microglial activation [301], thereby modulating immune responses. Together, these exRNAs exemplify how circulating RNA signatures in MS capture both pathogenic immune activation and loss of regulatory homeostasis.

In traumatic brain injury (TBI), exRNA release is rapid and dynamic, reflecting both primary mechanical damage and secondary injury cascades. Circulating damaged and oxidized RNAs increase acutely after injury, mirroring injury severity and, in some studies, predicting recovery trajectories [302,303,304,305]. Consistent with this, specific miRNAs such as miR-21 correlate with injury severity and neuroinflammatory responses [306], and miR-29a-5p has been shown to modulate blood–brain barrier integrity and NLRP3 inflammasome activation [307]. Collectively, MS and TBI illustrate how exRNA profiles can track both acute inflammatory states and chronic degenerative processes [302,303,304,305], albeit with differing temporal windows and biomarker requirements.

### 7.6. Glioblastoma and Other Brain Tumors

Brain tumors secrete abundant EVs containing tumor-specific RNAs that can be detected in CSF and, in some cases, plasma [111,308,309]. ExRNA profiling in glioblastoma has identified tumor-derived mRNAs, miRNAs, and mutant transcripts useful for diagnosis, molecular subtyping, and monitoring therapeutic response [310]. Because tumor EVs can dominate local exRNA pools [111,311], oncologic applications demonstrate the feasibility of cell-type enrichment and mutation-specific assays for sensitive detection [308,312].

### 7.7. Cross-Disease Patterns and Shared Pathways

**Figure 4:**
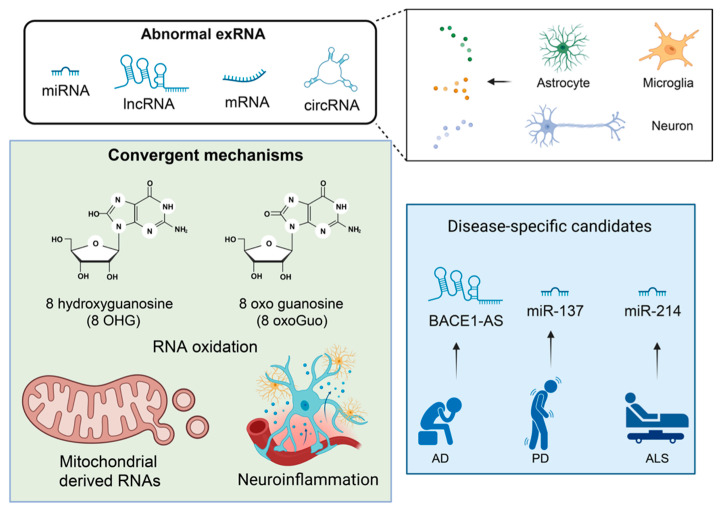
**Convergent and disease-specific exRNA-related mechanisms across neurodegenerative disorders**. This schematic summarizes shared and disease-specific extracellular RNA (exRNA) features across major neurodegenerative disorders, including Alzheimer’s disease (AD), Parkinson’s disease (PD), and amyotrophic lateral sclerosis (ALS). Abnormal exRNAs of diverse classes (miRNAs, lncRNAs, mRNAs, circRNAs) can be released and act within distinct neural cell types, including neurons, astrocytes, and microglia. Convergent mechanisms observed across diseases include RNA oxidation (e.g., 8-hydroxyguanosine and 8-oxo-guanosine), mitochondrial-derived RNAs, and neuroinflammatory signaling, reflecting shared stress and degenerative pathways. In parallel, disease-specific exRNA candidates (e.g., BACE1-AS in AD, miR-137 in PD, and miR-214 in ALS) illustrate how distinct molecular signatures may confer mechanistic specificity. This figure is intended as a conceptual integration of themes discussed in Section 7.7 rather than an exhaustive or quantitative comparison of individual exRNA species. Colored dots represent extracellular RNAs (exRNAs) originating from different CNS cell types, with green, orange, and blue indicating astrocyte-, microglia-, and neuron-derived exRNAs, respectively. Arrows indicate the directional association of exRNAs with downstream convergent mechanisms or disease-specific candidate pathways. Dashed lines, brackets, and dashed frames denote conceptual groupings or modular organization rather than direct physical interactions. Created in BioRender. Lu, K. (2025) https://BioRender.com/f0xubmk.

Across these disorders, common exRNA themes emerge: altered miRNAs regulating inflammation and mitochondrial pathways [13,108,207,250], elevated mitochondrial RNAs and oxidatively modified nucleic acids [72,98,108,109,110,287], and disease-associated lncRNAs that modulate pathogenic transcripts [210,250]. Also, across AD, PD, and ALS, human tissues and models consistently show elevated RNA oxidative modifications such as 8-hydroxyguanosine (8-OHG) and 8-oxo-guanosine (8-oxoGuo) [83], indicating that oxidative-damage-related nucleic acid signals in exRNAs could enable early detection of general neurodegenerative changes. In contrast, disease-specific candidates (for example BACE1-AS in AD [63], miR-137 in PD [67], miR-214 in ALS [152]) provide mechanistic specificity (Figure 4). Disentangling central from peripheral contributions and validating candidates across large, diverse, longitudinal cohorts remain key priorities to move exRNA markers toward clinical use (see Figure 1, Figure 3 and Figure 4 and Table 5).

### 7.8. Vascular Dementia and Other Underrepresented Conditions

Compared with neurodegenerative disease, exRNA studies in vascular dementia (VaD) remain relatively sparse. Available reports primarily implicate circulating and EV-associated miRNAs linked to endothelial dysfunction, inflammation, and blood–brain barrier disruption, reflecting the vascular origin of pathology [313,314]. For example, miRNAs involved in angiogenesis and vascular integrity have been reported to differ between VaD patients and controls, although most findings remain correlative and lack mechanistic validation [315]. These observations highlight both the potential and the current limitations of exRNA-based biomarkers in cerebrovascular cognitive disorders, underscoring the need for dedicated, well-powered studies in underrepresented disease contexts.

## 8. Translational Potential and Therapeutic Applications

Extracellular RNAs offer multiple translational opportunities spanning early detection, prognosis, treatment monitoring, and novel therapeutic strategies [274]. As minimally invasive liquid-biopsy analytes, exRNA panels could enable population screening to identify individuals at risk (for example those with MCI likely to progress to AD) [65] or to stratify patients for clinical trials of disease-modifying therapies [64]. Combining exRNA signatures with established modalities—neuroimaging, cerebrospinal fluid protein biomarkers, and clinical measures—can improve diagnostic accuracy and provide mechanistic context that informs patient selection and endpoint design [316].

For prognosis and monitoring, longitudinal exRNA measurements have the advantage of capturing dynamic transcriptional and metabolic shifts [65,317,318,319,320]. Candidate markers such as plasma PHGDH mRNA, mitochondrial transcripts, and specific miRNA panels have shown promise in predicting conversion or correlating with progression [64,65,72]. Real-time changes in exRNA profiles could therefore serve as proximal pharmacodynamic readouts in early-phase trials, indicating target engagement or metabolic rescue prior to changes in conventional imaging or functional endpoints [64,65,72,317].

Therapeutically, exRNAs themselves are both targets and vehicles [114,321,322]. Blocking maladaptive exRNA signaling—for instance, preventing release or uptake of vesicles enriched in neurotoxic miRNAs—represents a conceptually appealing strategy supported by proof-of-principle studies [323,324,325], but its translational feasibility remains constrained by limited BBB penetration, variable biodistribution, and off-target effects. Approaches include neutralizing antibodies against vesicle surface proteins [324,326], engineered decoy RNAs [327,328,329], antisense oligonucleotides to deplete specific extracellular lncRNAs [330,331,332], or small molecules that modulate vesicle biogenesis and release [333,334,335]. Preclinical models have suggested that perturbing EV production or specific RNA cargos can alter disease phenotypes [115,333,336], supporting further exploration.

Conversely, the delivery of therapeutic extracellular RNAs to the brain represents an attractive avenue for future neurodegenerative disease interventions [337,338]. However, the translational maturity of different RNA delivery platforms varies substantially, particularly in the context of CNS applications.

Engineered EVs bearing neuroprotective miRNAs, mRNAs, or antisense sequences have been shown to modulate neuronal and glial pathways following intrathecal administration or, in selected cases, after extensive surface engineering to facilitate BBB transit [339,340,341]. Although their endogenous origin and biocompatibility are appealing, systemic delivery studies consistently report limited brain accumulation [342,343,344], with efficient BBB crossing typically requiring substantial engineering [337]. Moreover, cargo loading efficiency, batch-to-batch heterogeneity, and off-target uptake by peripheral organs remain major unresolved challenges that currently constrain their translational scalability [345]. Lipid nanoparticles (LNPs), while highly efficient for systemic RNA delivery [346,347], exhibit pronounced liver tropism and negligible BBB penetration following intravenous administration and are frequently associated with innate immune activation [348,349]. These features substantially limit their direct applicability for CNS-targeted RNA delivery. Taken together, these considerations underscore that, although RNA-based delivery strategies are promising, no existing platform simultaneously achieves efficient BBB penetration, cell-type specificity, and minimal off-target effects, highlighting the need for careful platform selection and rigorous preclinical validation prior to clinical translation.

Diagnostic development faces regulatory and pragmatic hurdles. Analytical validity (assay reproducibility), clinical validity (sensitivity, specificity, predictive value), and clinical utility (impact on management and outcomes) must be demonstrated [280,350]. Multiplex classifiers combining exRNAs with other biomarkers will likely be necessary to reach clinically actionable performance, and prospective, multi-center studies with diverse populations are required to establish generalizability [351,352,353]. Cost, standardization of pre-analytical workflows, and integration into clinical pathways will ultimately determine real-world uptake.

In summary, exRNA biology creates a rich translational landscape: exRNAs can function as early, dynamic biomarkers for risk detection and monitoring, as targets whose neutralization may attenuate intercellular propagation of pathology, and as vehicles for delivering therapeutic RNAs [65,337,348]. Realizing these applications requires rigorous validation, standardized assays, safety evaluation, and carefully designed clinical studies that link molecular changes to meaningful clinical outcomes (see Figure 1, Figure 2 and Figure 3 and Table 3 for candidate markers, organ–brain axes, and schematic translational pathways.)

## 9. Limitations, Gaps and Recommendations

Despite growing promise, exRNA research in brain disorders faces substantial limitations that must be addressed to realize clinical translation. Key challenges include methodological heterogeneity, incomplete mapping of cellular origin and causality, limited cohort sizes and diversity, and regulatory and practical barriers to assay deployment [354,355,356].

Methodological heterogeneity and reproducibility: Pre-analytical variability (choice of biofluid, anticoagulant, time to processing, centrifugation, storage conditions) and divergent isolation methods (total plasma RNA, ultracentrifugation, size-exclusion chromatography, precipitation kits, immunocapture) produce inconsistent representations of vesicular versus non-vesicular RNA pools [13,14,15]. Profiling platforms (small-RNA vs. total RNA sequencing, RT-qPCR, ddPCR) and normalization strategies further complicate cross-study comparison [264,357]. To improve reproducibility, the field must adopt standardized standard operation procedures (SOPs) for sample collection and processing, report detailed metadata, characterize EV preparations (size distribution, protein markers), and use harmonized bioinformatic pipelines [272]. In this context, community-driven guidelines such as the Minimal Information for Studies of Extracellular Vesicles (MISEV) recommendations provide a practical framework for EV isolation, characterization, and reporting, and should be routinely referenced and implemented in exRNA studies [119].

Cellular origin and causal mechanisms: Many circulating exRNA signals are composite, reflecting CNS release, peripheral responses, or systemic metabolic changes [358,359]. Assigning cellular origin requires orthogonal evidence—CSF–blood comparisons, enrichment of neuron-derived EV markers, spatial or single-cell transcriptomics, and ideally in vivo tracing experiments. Establishing causality (distinguishing markers from mediators) demands perturbation studies in cellular and animal models combined with longitudinal human data; without such triangulation it is difficult to decide whether targeting a given exRNA will have therapeutic benefit.

Cohort size, diversity, and longitudinal data: Numerous reports rely on small, cross-sectional cohorts lacking replication. Robust biomarkers require adequately powered discovery cohorts, independent validation sets, and longitudinal sampling to test predictive value (for example MCI→AD conversion) and temporal stability [354,360]. Inclusion of diverse populations (age, sex, ethnicity, comorbidities) is essential to ensure generalizability and to avoid biased classifiers [354,360]. Future studies should therefore incorporate stratified sampling designs, pre-specified diversity targets, and transparent reporting of ancestry and sociodemographic variables, in line with broader clinical research inclusion policies.

Biological confounders and specificity: exRNA levels are influenced by comorbidities (metabolic disease, infection), medications, circadian factors, and recent activity, which can confound disease associations [229,246,361]. Additionally, shared pathways across neurodegenerative diseases (mitochondrial dysfunction, inflammation) produce overlapping exRNA signatures that can reduce diagnostic specificity [229,246,361]. Multiparametric panels and multimodal integration (proteomics, metabolomics, imaging) can improve specificity but increase complexity.

Technical sensitivity versus clinical robustness: Highly sensitive assays detect low-abundance RNAs but risk false positives from contamination or assay noise; conversely, stringent filters may miss biologically relevant low-abundance markers. Clinical assays must balance sensitivity with robustness, demonstrating repeatability across laboratories and conditions.

Regulatory, clinical, and ethical considerations: Translating exRNA assays to clinical use demands demonstration of analytical and clinical validity and clinical utility, along with regulatory approval [362]. Ethical issues include returning risk information for preclinical disease states, privacy concerns for molecular profiling, and equitable access to diagnostics [362].

Based on these gaps we recommend a roadmap: (1) community adoption of standardized pre-analytical and analytical SOPs and minimum reporting standards [179,264,363], including alignment with established EV-specific guidelines such as MISEV [119]; (2) routine characterization of EV and non-EV fractions and transparent method reporting [155,364]; (3) incorporation of cell-type enrichment and orthogonal tissue/CSF comparisons to strengthen origin assignment [245,365,366]; (4) large, multi-center longitudinal cohorts with diverse participants and harmonized protocols [364,366], potentially coordinated through international consortia such as the NIH Extracellular RNA Communication Consortium, which provide shared infrastructure, data standards, and community resources; (5) mechanistic perturbation studies to test causality and therapeutic potential [246,367,368]; (6) integration of exRNA data with proteomics, metabolomics, and imaging for multimodal classifiers [369,370,371]; and (7) early engagement with regulatory bodies to define evidence requirements for clinical deployment.

Addressing these gaps will be essential to move exRNA biomarkers from promising research findings to reliable clinical tools and to determine whether manipulating exRNA pathways can safely and effectively modify disease trajectories.

## 10. Conclusions and Future Perspectives

Extracellular RNAs occupy a unique link between cellular state and accessible peripheral measurement, offering a window into early transcriptional and metabolic disturbances that precede overt protein aggregation and structural decline in brain disorders [60,274,372,373]. Across AD (including MCI), PD, ALS, FTD, HD, MS, TBI, and glioblastoma, accumulating evidence shows that circulating and EV-associated RNAs (miRNAs, lncRNAs, circRNAs, mRNAs, and mt-RNAs) reflect disease-relevant pathways—mitochondrial dysfunction, oxidative stress, and neuroinflammation—and, in some settings, predict conversion or correlate with progression [61,62,63,64,65,66,67,72,83,151,152,153]. Mechanistic studies further indicate that exRNAs are not mere byproducts: they can be functionally transferred between neurons, glia, and peripheral cells to modulate synaptic integrity, immune tone, and metabolic homeostasis [12,17,181,195], supporting both diagnostic and therapeutic exploration.

Realizing the translational promise of exRNAs requires addressing methodological and conceptual hurdles. Standardized sample-handling protocols, transparent reporting of isolation and profiling methods, and routine characterization of EV and non-EV fractions are immediate technical priorities [13,14,15]. Demonstrating cellular origin and causality demands orthogonal approaches—CSF–blood comparisons, cell-type immunocapture, tissue correlation, spatial and single-cell transcriptomics, and perturbation experiments—to distinguish markers from mediators and to prioritize therapeutic targets [38,46,47,69,70,71]. Large, diverse, longitudinal cohorts with integrated multi-omics and imaging readouts are needed to validate candidate panels, assess predictive value, and ensure generalizability.

Near-term clinical applications are most likely to emerge from multimodal classifiers that combine exRNA features with established biomarkers (imaging, CSF proteins) to improve early detection and trial enrichment [57,274,374]. In parallel, preclinical and early-phase clinical studies should evaluate strategies to neutralize pathogenic exRNA signaling or to deliver therapeutic RNAs using engineered vesicles or lipid nanoparticles, with careful attention to biodistribution, immune safety, and target engagement [374,375,376].

In summary, exRNA research provides a powerful conceptual and technical framework to detect early brain dysfunction, monitor disease dynamics, and potentially intervene in the intercellular propagation of pathology [57,115,274,369,377,378]. By harmonizing methods, strengthening mechanistic evidence, and validating candidate markers in rigorous longitudinal studies, the field can move from promising discovery to robust clinical tools that enable earlier diagnosis, better prognostication, and novel RNA-based therapies for neurodegenerative and brain disorders.

## Data Availability

No new data were created or analyzed in this study. Data sharing is not applicable to this article.
